# Comparison of the Efficacy of Two Protocol Treatments in Patients with Symptomatic Disc Displacement without Reduction: A Randomized Controlled Trial

**DOI:** 10.3390/jcm12093228

**Published:** 2023-04-30

**Authors:** André Mariz de Almeida, João Botelho, Vanessa Machado, José João Mendes, Cristina Manso, Santiago González-López

**Affiliations:** 1 Clinical Research Unit (CRU), Egas Moniz Center for Interdisciplinary Research (CiiEM), Egas Moniz School of Health and Science, Caparica, 2829-511 Almada, Portugal; jbotelho@egasmoniz.edu.pt (J.B.); vmachado@egasmoniz.edu.pt (V.M.); jmendes@egasmoniz.edu.pt (J.J.M.);; 2 Sams Mais—Centro Clinico, 1070-128 Lisboa, Portugal; 3 School of Dentistry, Campus de Cartuja, University of Granada, Colegio Maximo s/n, 18011 Granada, Spain; sglopez@ugr.es; 4 Evidence-Based Hub, Egas Moniz Center for Interdisciplinary Research (CiiEM), Egas Moniz—Cooperativa de Ensino Superior, Caparica, 2829-511 Almada, Portugal

**Keywords:** arthrocentesis, hyaluronic acid, quality of life, temporomandibular joint, temporomandibular joint disorders, mandibular exercises

## Abstract

The aim of this study was to compare the effectiveness of arthrocentesis followed by hyaluronic acid infiltration treatment (ASH) and mandibular exercise therapy (MET) in patients with symptomatic disc displacement without reduction (DDwoR) by examining pain intensity (VAS), mandibular range of motion (MO), and quality of life (QoL). Fifty-two patients were randomly allocated into two groups, MET (N = 26) and ASH (N = 26), and therapy was applied at the baseline and one month after. Patients were followed up at 1 and 12 months after the baseline assessment. Clinical and patient-reported outcomes were compared at the baseline, 1-month follow-up, and 12-month follow-up. The study found no significant differences in VAS and MO between the ASH and MET groups at the baseline. However, while not significant, it was noted that the ASH group showed higher values for MO. Regarding OHIP-14 at 1 month of follow-up, the ASH group showed significant improvements in physical pain (*p* > 0.01), physical and psychological disability (*p* = 0.043 and *p* = 0.029), and handicap (*p* = 0.033). At the 12-month follow-up, the ASH group showed significant improvements in functional limitation, psychological discomfort, psychological disability, and handicap (*p* = 0.008, *p* = 0.001, *p* = 0.001, *p* = 0.005, respectively). ASH treatment did not reduce pain or improve mandibular range of motion more than physical therapy in patients with symptomatic DDwoR. However, ASH could be preferable given its positive long-term effects on patients’ quality of life. The clinician’s main objective is to prioritize the treatment plan order with a focus on the patient’s quality of life. Accordingly, healthcare professionals should consider ASH as a treatment option for patients with symptomatic DDwoR who desire long-term improvement in their quality of life.

## 1. Introduction

Intra-articular disorders of the temporomandibular joint (TM) are defined as an abnormal positional relationship between the disc and the condyle, articular eminence, and/or articular fossa [1]. The classification of TM disorders (TMD) is crucial for appropriate diagnosis and management of this complex condition. Schiffman et al. (2014) proposed a classification system [2]—Diagnostic Criteria for Temporomandibular disorders—that divides TMD into three main groups based on etiology: Group I, muscle disorders (including myofascial pain with and without mouth-opening limitation); Group II, disc displacement disorders (including disc displacement with or without reduction and mouth-opening limitation); and Group III, joint disorders (including arthralgia, arthritis, and arthrosis). TMD is a prevalent condition that affects the temporomandibular joint and associated structures, causing pain and functional limitations. According to an evidence-based study, TMD is the second most common musculoskeletal disorder that causes pain and disability [3]. The study reported a pooled prevalence of TMD of 18.6%, with higher rates in women and older adults. Jin et al. (2016) also highlighted the global burden of oral diseases, including TMD, and the need for effective management strategies [4]. These findings emphasize the importance of TMD as a public health concern and the need for appropriate diagnosis and management to improve patients’ quality of life. TMD is frequently comorbid with other conditions, including fibromyalgia and headaches. The underlying mechanism of this comorbidity is thought to be central sensitization, a process in which the central nervous system becomes hypersensitive to sensory stimuli [5,6]. Recently, studies have utilized advanced techniques, such as machine learning, to further investigate the relationship between TMD, neck pain, and primary headaches. They revealed that central sensitization symptoms and psychosocial alterations may be key factors in the comorbidity between TMD and primary headaches, indicating the need for a more comprehensive approach to the diagnosis and treatment of these complex conditions.

Disc displacement with reduction is the most prevalent diagnosis among the intra-articular disorders of the TMJ, corresponding to 41% of TMD clinical diagnoses [7,8]. On the other hand, disc displacement without reduction (DDwoR) incidence among TMD patients is estimated to be between 2% and 8% [9,10]. The etiology of DDwoR is unclear; however, anatomical factors, parafunctions, trauma, or hypermobility of the joints have all been reported to play a key role in structural changes to the TMJ, which could result in disc displacements [11,12,13,14]. 

Clinically, the main symptoms of DDwoR are characterized by TMJ pain and limited mouth opening (“closed lock”), which result from the overload of the bilaminar zone by direct contact with the mandible’s head and the mechanical blocking of the translation movement of the mandible’s head due to the displacement of the disc anteriorly, respectively [15,16]. Additionally, it has been reported that the natural course of DDwoR and the closed lock accompanying a DDwoR is self-limiting and favorable for most patients [16,17,18,19]. However, it is also well stated that internal derangements of the TMJ such as DDwoR may lead to osteoarthritis [20].

Various interventions have been suggested for DDwoR, but, to date, the most effective approach is still unclear. Primary treatment options include conservative, non-surgical treatment modalities, such as mandibular manipulation technique, education and counseling, splints, pharmacotherapy, and physical therapy [16,21]. Exercise therapy is part of the physical treatment and includes active exercises (correct mouth-opening path) and passive exercises (improve mouth-opening range) [16]. Studies assessing the efficacy of exercise therapy in DDwoR showed a reduction in pain and an improvement in the mandible’s range of motion (opening, protrusion, and lateral movements) after applying an algorithm of exercise therapies [16,17,21]. It should be noted that, due to the heterogeneity of these studies, their results should be interpreted with caution [22]. 

Minimally invasive therapies, which include arthrocentesis (AT), are the second treatment option when conservative therapies are not effective in reducing signs and symptoms of DDwoR [21,23]. In TMJ arthrocentesis, the upper joint space is irrigated by introducing one or two needles to achieve throughflow of fluid (saline solution) and lavage of the joint [24,25]. Systematic reviews have reported that TMJ arthrocentesis improves jaw function and reduces pain levels, and the execution of multiple sessions is superior to a single session [25,26,27]. Its effectiveness could be explained by the joint space expansion achieved, the breaking of joint adhesions with the introduction of fluid, and the washing out of inflammatory mediators and catabolites [26,27,28]. Certain studies propose that first-line TMJ arthrocentesis has a variable and insufficient effect, resulting in either a significant or only a small decrease in pain scores, and either way with no improvement in mandibular movements when compared with conservative treatments. These results should be taken with caution since they come from a small number of trials with high heterogeneity, but nonetheless they cast doubt on the effectiveness of AT [27]. In an effort to improve its effectiveness, the additional use of sodium hyaluronate (SH) after an AT procedure has been proposed for the improvement of patients’ outcomes [26]. Overall, the current evidence does not provide strong support for the effectiveness of AT when compared with conservative modalities in DDwoR and osteoarthritis. 

Thus, we conducted a randomized clinical trial to assess the efficacy of AT compared with physical therapy in patients with DDwoR with osteoarthritis, aiming to test the hypothesis that AT is more effective than physical therapy. 

## 2. Materials and Methods

This was a double-arm randomized controlled trial approved by the institutional review board at the SAMS Hospital (ID-012018) and registered in ISRCTN (number: 48852162) [29], conducted from March 2019 to July 2021, according to the Declaration of Helsinki of 1975. All subjects received a detailed explanation of the study and signed a written informed consent form prior to the beginning of the study.

### 2.1. Participants

Patients with DDwoR with osteoarthritis treated at or referred to the orofacial pain clinic of Centro Clinico SAMS Serviços de Assistência Medico Social were recruited.

Inclusion criteria were age between 18 and 70 years, uni- or bilateral DDwoR diagnosis according to the Portuguese version of the Diagnostic Criteria for Temporomandibular Disorders (DC/TMD) [30] by one calibrated researcher (kappa coefficient = 0.80), previous history of limited mouth opening due to TMJ blocking, pain in the TMJ for at least 6 months, and previous treatments. There was no sex restriction. Exclusion criteria were exclusively muscular TMD diagnosis, fibromyalgia and systemic inflammatory diseases, dental and neuropathic pain, clinical history of TMJ fracture, ankylosis or surgery, and ongoing articular TMD or orthodontic treatment. 

After applying the inclusion criteria, computerized tomography and magnetic resonance imaging was performed on the included patients to confirm the presence of DDwoR and osteoarthritis.

### 2.2. Study Protocol, Randomization, and Blinding

**Figure 1:**
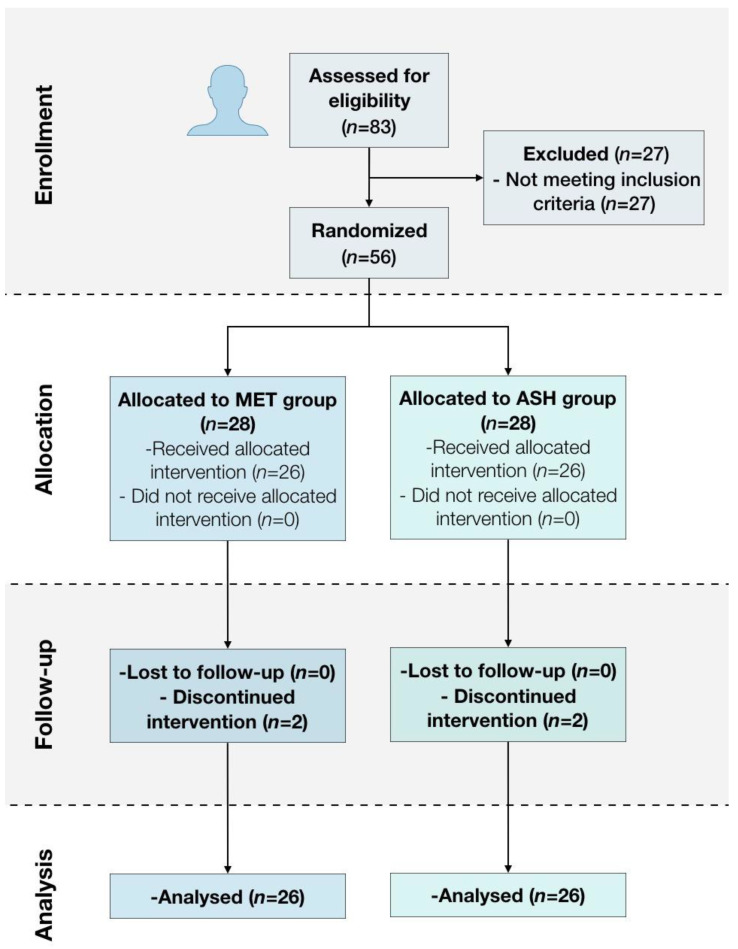
Flow diagram of patient enrollment, allocation, and follow-up.

Patients were seen on 4 occasions. During the first visit, patients were screened for inclusion in the study. Patients were then informed of the treatments and assessment tools used in the study. Patients were told that they could receive mandibular exercise therapy (MET, *n* = 26) or arthrocentesis and saline hyaluronate (ASH, *n* = 26). On the day of treatment administration (visit 2), participants were consecutively allocated (1:1) into treatment groups using a randomization tool (https://www.randomizer.org/, accessed on 10 February 2023) prior to procedures administered by a technician who was not involved in any other procedures in the study. Opaque envelopes were used to achieve allocation concealment. The envelopes were opened by the clinician immediately prior to treatment administration. The order of the envelopes was determined independently prior to the start of the study by an investigator who was not involved in the study. Patients were assessed for pain and functional measures immediately prior to the interventions, and treatment was administered thereafter. Follow-up visits were scheduled at 1 month (visit 3) and 12 months (visit 4). At the beginning of each follow-up visit, patients were assessed for pain and function using the same protocol as at the baseline. Interim phone calls were made at 15 days and 10 months post-treatment to improve retention and to monitor for adverse events. Figure 1 shows the flowchart of patient enrollment, allocation, and follow-up. 

### 2.3. Interventions

#### 2.3.1. Mandibular Exercise Therapy (MET Group)

The following set of home exercises were taught and demonstrated to the patients: (a) opening and closing the mouth with the tongue on the palate (10 repetitions); (b) moving the mandible right and left laterally with the index finger placed between the incisor teeth, which serve as a guidance platform (10 repetitions); and (c) blowing through the lips, causing the cheeks to vibrate during exhalation (10 repetitions). The exercises were always taught to the participants by the same physiotherapist. Patients were required to perform the set of mandibular exercises five times throughout the day for 1 month, always in the same sequence. In order to dispel doubts about the exercises and to ask if they were being carried out, patients were contacted by telephone fortnightly by the physiotherapist. 

#### 2.3.2. Arthrocentesis + Sodium Hyalorante (ASH)

First, a straight line was drawn with a marker pen along the skin from the middle portion of the auricular tragus to the lateral corner of the eyeball. A single point located 10 mm from the tragus and 2 mm below the tragal–canthal line was marked for the insertion of the needle. Next, antisepsis of the whole face was performed with 2% chlorhexidine solution, with an emphasis on the pre-auricular region and the ear. Then, both auriculotemporal nerve block and masseteric and posterior deep temporal nerve block were conducted with lidocaine. A 19 G needle connected to a 5 mL syringe was inserted into the marked point. When in place, 5 mL of saline solution at 0.9% was administered 5 times (20 mL of saline solution in total) in order to distend the joint space, and patients were instructed to open and close their mouths during this procedure to promote wash out of the cavity [28]. At the end of the procedure, 1 mL of SH (Osteonil Plus^®^ (TRB Pharma, Genève, Switzerland)—1.7 kDa) was injected into the upper TMJ compartment. Then, a mandibular maneuver physiotherapy session was conducted by the same physiotherapist. The following intraoral mobilization techniques were applied in the same sequence for 10 min: condylar distraction (mandibular distraction), bilateral (15 repetitions on each side); condylar laterality, bilateral (15 repetitions on each side); and anterior mandible translation (anterior translation mobilization), bilateral (15 repetitions on each side). After this mobilization sequence, participants were asked to perform five maximum mouth openings. The described procedures were performed at the baseline and at the 1-month follow-up.

### 2.4. Outcomes

Variables were assessed at the baseline and again at 1 and 12 months after treatment by a calibrated researcher who was not involved in any other procedure of the study.

#### 2.4.1. Pain Intensity (VAS)

Patients rated their pain intensity in the TMJ using a 100 mm visual analog scale (VAS) with endpoints “no pain” and “worst pain”. Participants were instructed to make a mark on the VAS indicating their level of pain at each visit [31]. Changes in average pain scores after treatment served as the primary outcome.

#### 2.4.2. Oral Health-Related Quality of Life (OHRQoL)

To assess OHRQoL, patients completed the validated Portuguese version of the Oral Health Impact Profile 14 (OHIP-14) [32]. Specifically, the OHIP-14 comprises fourteen questions of seven domains of OHRQoL (functional limitation, physical pain, psychological discomfort, physical disability, psychological disability, social disability, and handicap). Each question is scored on a 0–4 Likert scale (0—never; 1—hardly ever; 2—occasionally; 3—fairly often; and 4—very often). We also combined all scores from all questions into an overall score (minimum of 0 and maximum of 56), while domain values ranged from 0 to 8. 

#### 2.4.3. Mouth Opening (MO)

Pain-free mouth opening and maximum unassisted opening were measured with patients seated in a dental chair, in a room with adequate lighting, following the instructions of the DC/TMD-Axis I [30] using a plastic ruler (Therabite, Atos Medical^®^, Horby, Sweden). 

### 2.5. Statistical Methods

Considering a standard deviation of 1.0 for a 0.89 mean difference [33] and an 80% power with a 5% two-sided significance level, a minimum sample of 22 patients per arm was calculated. Considering a possible 30% dropout rate, a final number of 26 participants per group was defined as the minimum-required sample per group. 

The statistical approach was based on the site as a unit. Data analysis was performed using R for Macintosh (v4.0.1). An explicit comparison of mean values was performed using the Kruskal–Wallis test, with Benjamini–Hochberg adjustment used for multiple comparisons, as data assumptions for the application of the test did not meet normality and homoscedasticity. A chi-square test was used for comparisons of categorical variables across the groups. The level of statistical significance was set at 5% in all inferential analyses. 

### 2.6. Changes to Protocol

This trial was initially based on three different arms; however, the third intervention arm (viscosupplementation without arthrocentesis) was deemed impossible to carry out due to lack of available materials and funding.

## 3. Results

### 3.1. Patients’ Characteristics

Overall, 83 patients were screened; 27 patients were excluded for not meeting the inclusion criteria, and 56 eligible patients were enrolled in this study (mean age ± 49.4). Included patients were divided in 2 groups, each with 28 patients; however, 2 patients from each group did not complete the study due to discontinued interventions (Figure 1).

There were no differences in demographic characteristics regarding age, sex, education level, and DC/TMD diagnoses between treatment groups (Table 1). In the MET group, 20 participants had a unilateral DDwR with myalgia, while 6 had bilateral DDwR with myalgia. In the ASH group, 22 participants had a unilateral DDwR with myalgia, while 4 had bilateral DDwR with myalgia.

### 3.2. Pain Intensity (VAS)

Baseline data showed no significant inter-group differences (*p* > 0.05) in pain intensity (VAS) in post-treatment follow-ups (Table 2). 

### 3.3. Oral Health-Related Quality of Life (OHIP-14)

Regarding OHIP-14 baseline data, a significant improvement was found in the inter-group comparisons for physical pain and disability (*p* > 0.01; *p* > 0.043), psychological disability (*p* > 0.029), and handicap domains (*p* > 0.033) for the ASH group at the 1-month follow-up (Table 3). Additionally, a significant improvement was also found for the ASH group at the 12-month follow-up for functional limitation (*p* < 0.008), psychological discomfort and disability (*p* < 0.001; *p* < 0.001), and handicap (*p* < 0.005) (Table 3).

### 3.4. Mouth Opening (MO)

Considering the pain-free mouth opening and maximum unassisted opening, no significant differences were found between groups at baseline (*p* < 0.05) or in treatment follow-ups (Table 4). However, when comparing the improvement of groups considering baseline data and follow-ups, ASH presented higher values for both pain-free mouth opening and maximum unassisted opening (Table 4).

## 4. Discussion

Our hypothesis, which proposed that AT treatment for symptomatic DDwoR would be more effective than physical therapy as conservative treatment, could be partially confirmed through this study. Our findings demonstrate that, even though there were no significant differences between groups regarding pain intensity and mandibular range of motion (opening), all of the assessed domains related to the patients’ quality of life were significantly improved in the AT group.

When we consider the pain intensity results, our study showed that there was a decrease in subjective pain in both groups throughout the study, with no significant differences between them. It was very interesting to note that, despite the fact that our studied population was a refractory chronic sample, physical therapy, which is a conservative treatment and generally the first-line treatment, was as effective as AT. Similarly, mandibular opening increased in both groups in all follow-ups, with no differences between groups. These results align with a previous study demonstrating that both treatments are efficacious in diminishing subjective pain and improving mouth opening in patients with symptomatic DDwoR [34]. To improve on the current research results, it is important for future studies to test the use of AT during the first three months of physical therapy as conservative treatment in refractory chronic patients [35]. 

The fact that during AT there is a lavage and aspiration of inflammatory fluid mainly composed of proinflammatory cytokines, a joint space expansion, and breaking of joint adhesions could explain its positive effects in the outcomes assessed in this study [27]. In another study, the infiltration of SH after the AT procedure certainly played an important role in diminishing pain by equilibrating the homeostasis of TMJ [36]. Nevertheless, it is quite difficult to compare the physical therapy protocol proposed in our study with other protocols since there is a wide range of exercises that could improve mouth opening and decrease subjective pain [16,17]. The positive effects of physical therapy on mouth opening and subjective pain could be explained by displacement of the disc, which acted as a mechanical obstacle for mouth opening, and by the development of a hard region in the retrodiscal tissue, which is highly sensitive. Furthermore, we cannot exclude the fact that improvements in patients’ symptoms occurred regardless of the intervention modality, due to either the favorable, naturally self-limiting course of DDwoR or to placebo effects [37]. 

It is well known that TMD patients, independent of the diagnosis, present psychosocial impairment and a reduction in well-being (impaired quality of life) [38,39]. It is noteworthy that our study found an improvement in the quality of life in the ASH group alone in all domains at different periods of follow-up. Our findings align with the study of Castaño-Joaqui et al. [40], which found an improvement in OHIP-14 score after arthrocentesis plus saline hyaluronate treatment and concluded that self-perceived quality of life should be considered stable in the long term for the treatment to be regarded as successful. Our study assessed the quality of life of patients after 12 months and found that almost all domains of OHIP-14, except physical pain and disability, were stable. The change in physical pain and disability found in our study after one month of follow-up (improvement in subjective pain and mandibular range of motion) suggests an early relief of physical symptoms, and the progressive improvement in psychological disability could indicate that patients’ concerns about TMD pain improved in the short term and remained low in the long term. This demonstrates the use of carrying out an assessment of an individual’s well-being since data related to physical impairments, functional limitations, and general health status are important variables to assess the treatment effects.

Limitations of the present study include the potential regression to the mean and the natural disease course of DDwoR, as well as the lack of blinding of patients and researchers and the restricted studied population, with no gender pairing. Randomized controlled clinical trials including groups with only SH, arthrocentesis, and conservative therapies, assessing the efficacy and effects of this treatment on somatosensory variables of symptomatic DDwoR patients, are encouraged in the future. 

## 5. Conclusions

In view of the results and limitations of this study, it can be concluded that ASH treatment is not superior to physical therapy with regard to diminishing pain intensity and improving mandibular range of motion in patients with symptomatic DDwoR. However, ASH could be the preferred treatment for this diagnosis due to its long-term positive effects on patients’ quality of life.

## Figures and Tables

**Table 1:** Participants’ characteristics.

	MET (*n* = 26)	ASH (*n* = 26)	*p*-Value
Age, mean (SD)	48.6 (14.3)	47.3 (18.3)	0.964
Sex, *n* (%)			
Females	22 (84.6)	16 (61.5)	0.118
Males	4 (15.4)	10 (38.5)	
Education, *n* (%)			
Elementary	1 (3.8)	5 (19.2)	0.217
Middle	2 (7.7)	10 (38.5)	
Higher	2 (7.1)	7 (25.0)	
DDwR, *n* (%)			
Unilateral plus myalgia	20 (76.9)	22 (84.6)	0.482
Bilateral plus myalgia	6 (23.1)	4 (15.4)	

**Table 2:** Average facial pain intensity (VAS—mm) and inter-group differences assessed at different time points.

	Periods	MET (*n* = 26)	ASH (*n* = 26)	*p*-Value
	Baseline	6.8 (2.3)	6.7 (2.2)	0.648
VAS (mm)	1 month	5.0 (2.4)	3.3 (1.2)	0.328
	12 months	3.0 (2.5)	2.4 (2.1)	0.355

**Table 3:** Mean (± standard deviation) of OHIP-14 domain scores at different time points. * *p* > 0.05.

OHIP-14, Mean (SD)	MET (*n* = 26)	ASH (*n* = 26)	*p*-Value
Functional Limitation			
Baseline	2.6 (1.8)	2.4 (2.0)	0.597
1 month	1.4 (1.4)	1.1 (1.1)	0.600
12 months	1.5 (1.1)	0.7 (0.7)	0.008 *
Physical Pain			
Baseline	6.4 (1.3)	5.8 (1.4)	0.116
1 month	4.9 (2.0)	3.3 (1.2)	0.001 *
12 months	4.5 (2.0)	3.6 (1.4)	0.120
Psychological Discomfort			
Baseline	5.3 (1.7)	4.6 (2.0)	0.246
1 month	2.9 (2.2)	2.2 (1.6)	0.253
12 months	3.7 (2.1)	1.7 (1.4)	<0.001 *
Physical Disability			
Baseline	4.9 (1.8)	5.4 (1.6)	0.270
1 month	4.0 (2,1)	2.3 (1.6)	0.002 *
12 months	3.6 (2.4)	2.5 (1.7)	0.101
Psychological Disability			
Baseline	4.2 (1.8)	3.2 (1.8)	0.024
1 month	2.9 (1.8)	1.8 (1.5)	0.029
12 months	3.2 (1.9)	1.5 (1.0)	<0.001 *
Social			
Baseline	3.9 (2.0)	3.3 (2.1)	0.460
1 month	2.3 (1.9)	1.2 (1.4)	0.253
12 months	2.7 (1.9)	1.4 (2.1)	<0.001 *
Handicap			
Baseline	3.0 (1.9)	2.2 (1.4)	0.131
1 month	1.9 (1.5)	1.0 (0.8)	0.033
12 months	2.3 (1.6)	1.1 (1.0)	0.005 *

**Table 4:** Mean and standard deviation of pain-free mouth opening and maximum unassisted opening (mm) in different evaluation periods. * *p* > 0.05.

DC/TMD—Axis I	MET (*n* = 26)	ASH (*n* = 26)	*p*-Value
Pain-free opening			
Baseline	33.6 (6.2)	32.6 (5.9)	0.378
1 month	37.2 (5.9)	38.8 (4.5)	0.254
12 months	37.5 (5.0)	39.5 (4.7)	0.125
Difference from baseline			
At 1 month	3.7 (3.1)	6.2 (4.4)	0.007 *
At 12 months	3.9 (3.4)	6.9 (4.5)	0.016 *
Maximum unassisted opening			
Baseline	37.1 (5.5)	36.0 (4.9)	0.239
1 month	39.5 (5.1)	41.4 (4.0)	0.142
12 months	39.8 (4.6)	41.7 (4.2)	0.101
Difference from baseline			
At 1 month	2.4 (2.5)	5.4 (3.2)	0.003 *
At 12 months	2.4 (2.5)	5.6 (3.7)	0.008 *

## Data Availability

Not applicable.
